# Quantum Neural Network Based Machine Translator for Hindi to English

**DOI:** 10.1155/2014/485737

**Published:** 2014-02-27

**Authors:** Ravi Narayan, V. P. Singh, S. Chakraverty

**Affiliations:** ^1^Department of Computer Science & Engineering, Thapar University, Patiala, Punjab 147 004, India; ^2^Department of Mathematics, National Institute of Technology Rourkela, Odisha 769 008, India

## Abstract

This paper presents the machine learning based machine translation system for Hindi to English, which learns the semantically correct corpus. The quantum neural based pattern recognizer is used to recognize and learn the pattern of corpus, using the information of part of speech of individual word in the corpus, like a human. The system performs the machine translation using its knowledge gained during the learning by inputting the pair of sentences of Devnagri-Hindi and English. To analyze the effectiveness of the proposed approach, 2600 sentences have been evaluated during simulation and evaluation. The accuracy achieved on BLEU score is 0.7502, on NIST score is 6.5773, on ROUGE-L score is 0.9233, and on METEOR score is 0.5456, which is significantly higher in comparison with Google Translation and Bing Translation for Hindi to English Machine Translation.

## 1. Introduction

Machine translation is one of the major fields of NLP in which the researchers are having their interest from the time computers were invented. Many machine translation systems are available with their pros and cons for many languages. Researchers have also presented different approaches for computer to understand and generate the languages with semantics and syntactics. But still many languages are having translation difficulties due to ambiguity in their words and the grammatical complexity. The machine translator should address the key characteristic properties which are necessary to increase the performance of machine translation up to the level of human performance in translation. Most of the machine translators are working on the alignment of words in chunk (sentence).

This paper presents the quantum neural based machine translation for Hindi to English. The quantum neural network (QNN) based approach increases the accuracy during the knowledge adoptability. In this work our main focus is to show the significant increase in the accuracy of machine translation during our research with the pair of Hindi and English sentences. The machine translation is done using the new approach based on quantum neural network which learns the patterns of language using the pair of sentences of Hindi and English.

Some researchers have done their machine translation (MT) using statistical machine translation (SMT). The SMT uses the pattern recognition for automatic machine translation systems for available parallel corpora. Statistical machine translation needs alignment mapping of words between the source and target sentence. On one hand alignments are used to train the statistical models and, on the other hand, during the decoding process to link the words in the source sentence to the words of target sentence [1–4]. But SMT methods are having the problem of word ordering. To overcome the problem of word ordering and for increasing the accuracy, some researchers introduced the concept of syntax-based reordering for Chinese-to-English and Arabic-to-English [5].

Recently some work has been done with Hindi by several researchers using different methods of machine translation, like example based system [5, 6], rule based [7], statistical machine translation [8], and parallel machine translation system [9]. A. Chandola and Mahalanobis described the use of corpus pattern for alignment and reordering of words for English to Hindi machine translation using the neural network [10], but still there are a lot of possibilities to develop a MT System for Hindi to increase the accuracy of MT. Some of the important works on Hindi are discussed in Section 2.

The main motivation behind the study of QNN is the possibility to address the unrealistic situation as well as realistic situation, which is not possible with the traditional neural network. QNN learns and predicts more accurately and needs less computation power and time for learning in comparison to artificial neural network. Researchers introduced the novel approach of neural network model based on quanta states superposition, having multilevel transfer function [11–13].

The most important difference among classical neural network and QNN is of their respective activation functions. In QNN as a substitute of normal activation functions, a multilevel activation function is used. Each multilevel function consists of the summation of sigmoid functions excited with quantum difference [14].

In QNN, the multilevel sigmoid function has been employed as activation function and is expressed as

(1) $$\begin{array}{c} \text{sgm}\left(x\right)=\frac{1}{n_{s}}\sum_{r=1}^{n_{s}}\left(\frac{1}{1+\exp\left(x-\theta^{r}\right)}\right), \end{array}$$

where *n*_*s*_ denotes the total multilevel positions in the sigmoid functions and *θ* ^*r*^ denotes quantum interval of quantum level *r* [2].

## 2. Hindi-English Machine Translation Systems 

### 2.1. ANGLABHARTI-II Machine Translation System

ANGLABHARTI-II was proposed in 2004 which is a hybrid system based on the generalized example-base (GEB) with raw example-base (REB). At the time of development, the author establishes that the alteration in the rule-base is hard and the outcome is possibly random. This system consists of error-analysis component and statistical language component for postediting. Preediting component can change the entered sentence to a structure, to translate without difficulty [5].

### 2.2. MATRA Machine Translation System

The MaTra was introduced in 2004 which is based on transfer approach using a frame-like structured representation. In this the rule-based and heuristics approach is used to resolve ambiguities. The text classification module is used for deciding the category of news item before working in entered sentence. The system selects the appropriate dictionary based on domain of news. It requires human assistance in analyzing the input. This system also breaks up the complex English sentence to easy sentences, after examining the structure, it produces Hindi sentences. This system is developed to work in the domain of news, annual reports, and technical phrases [7].

### 2.3. Hinglish Machine Translation System

Hinglish machine translation system is developed in 2004 for standard Hindi to standard English. This system is developed to incorporate the added enhancement to available AnglaBharti-II MT System for English to Hindi and to AnuBharti-II systems for Hindi to English translation, developed by Sinha. The accuracy of this system is satisfactory more than 90%. As the verbs have multiple meanings, it is not able to determine the sense, due to nondeep grammatical analysis [6].

### 2.4. IBM-English-Hindi Machine Translation System

IBM-English-Hindi MT System is developed by IBM India Research Lab in 2006; at the beginning of this project they started to develop an example based MT system but later on shifted to the statistical machine translation system from English to Indian languages.

### 2.5. Google Translate

Google Translate was developed by Franz-Josef Och in 2007. This model used the statistical MT approach to translate English to other languages and vice versa. Among the 57 languages, Hindi and Urdu are the only Indian languages present with Google Translate. Accuracy of the system is good enough to understand the sentence after translation [15].

## 3. Proposed Machine Translation System for Hindi to English

**Figure 1:**
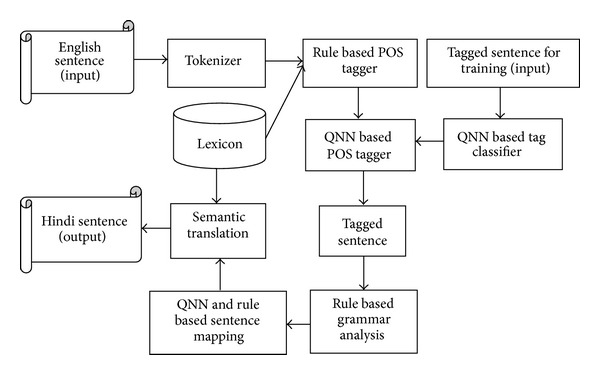
Architecture of MT system model.

The proposed machine translation (MT) system consists of two approaches, one is rule based MT system and the other is quantum neural based MT system. The source language goes into the rule based MT system and passes through the QNN based MT system to refine the MT done by rule based MT module, which basically recognizes and classifies the sentence category. 2600 sentences are used with English and their corresponding Devanagari-Hindi sentences. Each Devanagari-Hindi sentence consists of words with question word, noun, helping verb, negative word, verb, preposition, article, adjective, postnoun, adverb, and so forth. Each English sentence contains a question word, noun, helping verb, negative word, verb, preposition, article, adjective, postnoun, adverb, and so forth. The data used to train is produced by an algorithm, which is based on simple deterministic grammar. The entire architecture of the proposed MT system model is given in Figure 1.

## 4. Quantum Neural Architecture 

**Figure 2:**
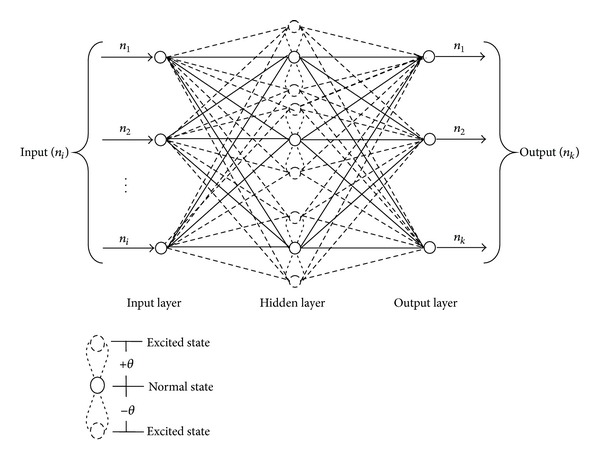
Architecture of Quantum Neural Network.

As shown in the Figure 2, three-layer architecture of QNN consist of inputs, one layer of multilevel hidden units, and output layer. In QNN as a substitute of normal activation functions, a multilevel activation function is used. Each multilevel function consists of summation of sigmoid functions excited with quantum difference.

Where *n*_*s*_ denotes total multilevel positions in sigmoid functions, *θ* ^*r*^ denotes quantum interval of quantum level *r*:

(2) $$\begin{array}{c} \text{sgm}\left(x\right)=\frac{1}{n_{s}}\sum_{r=1}^{n_{s}}\left(\frac{1}{1+\exp\left(-x\pm \theta^{r}\right)}\right). \end{array}$$

Here every neural network node represents three substates in itself with the difference of quantum interval *θ* ^*r*^ with quantum level *r*, where *n*_*s*_ denotes the number of grades in the quantum activation functions.

## 5. Quantum Neural Implementation of Translation Rules

The strategy is to first identify and tag the parts of speech using Table 1 and then translate the English (source language) sentences literally into Devanagari-Hindi (target language) with no rearrangement of words. After syntactic translation, rearrangement of the words has been done for accurate translation retaining the sense of translated sentence. The rules are based on parts of speech, not based on meaning. To facilitate the procedure, distinctive three-digits codes based on their parts of speech are assigned which are shown in Table 1.

For a special case when input sentence and the resulting sentence are having unequal number of words, then the dummy numeric code .000 is used for giving a similar word alignment.

**Case 1**

When input sentence and the resulting sentence are having unequal numbers of words.

The coded version of sentence is thus

 Ram will  not   go  to the market.
 .100 .111   .220 .110  .140  .123  .102

Therefore, input numeral sequence is [.100.111.220.110.140.123.102] and the corresponding output is [.100.102.220.110.111.000.000]. The outcome of neural network might not be the perfect integer, it should be round off and few basic error adjustments might be needed to find the output numeral codes. Even the network is likely to arrange the location of 3-digit codes. By this, it learns the target language knowledge which is needed for semantic rearrangement and also helps in parts of speech tagging, by pattern matching: it is also helpful to adopt and learn the grammar rules up to a level. For handling the complex sentences the algorithm is used. The algorithm first removes the interrogative and negative words, on the basis of conjunction; the system breaks up and converts the complex sentence into two or more small simple sentences. After the translation of each of the simple sentences, the system again rejoins the entire subsentences and also adds the removed interrogative and negative words in the sentence. The whole process is explained in Algorithm 1 in the next section.

### 5.1. Algorithm for Proposed QNN Based MT System for Complex Sentences

*QNNMTS (SENTENCE, TOKEN, N, LOC).* Here SENTENCE is an array with *N* elements containing Hindi words. Parameter TOKEN contains the token of each word and LOC keeps track of position. ICOUNT contains the maximum number of interrogative words encountered in sentence, NCOUNT contains the maximum number of negative words encountered in the sentence, and CCOUNT contains the maximum number of conjunction words encountered in the sentence. (see Algorithm 1).

## 6. Experiment and Results

All words in each language are assigned with a unique numeric code on the basis of their respective part of speech. Experiments show that memorization of the training data is occurring. The results shown in this section are achieved after training with 2600 Devanagari-Hindi sentences and their English translations. 500 tests are performed with the system for each value of quantum interval (*θ*) with random data sets selected from 2600 sentences; the dataset is divided in 4 : 3 : 3 ratios,respectively, for training, validation, and test from 2600 English sentences and their Devanagari-Hindi translations. In Table 2, the values are the average of 500 tests performed with the system for each value of quantum interval (*θ*) for 2600 sentences. The best performance is shown for value of quantum interval (*θ*) equal to one with respect to all the parameters; that is, epoch or iterations needed to train the network, the training performance, validation performance, and test performance in respect to their mean square error (MSE). Here it is clearly shown that QNN at (*θ*) equal to one is very much efficient as compared to classical artificial neural network at (*θ*) equal to zero. Table 2 clearly shows the comparison between the performances of QNN with ANN in respect to above said performance parameters and as a result we can conclude that QNN is better than ANN for machine translation.

## 7. Evaluations and Comparison

This paper proposed a new machine translation method which can combine the advantage of quantum neural network. 2600 sentences are used to analyze the effectiveness of the proposed MT system.

The performance of proposed system is comparatively analyzed with Google Translation (http://translate.google.com/) and Microsoft's Bing Translation (http://www.bing.com/translator) by using various MT evaluation methods like BLEU, NIST, ROUGE-L, and METEOR. For evaluation purpose we translate the same set of input sentences by using our proposed system, Google Translation, and Bing Translation and then evaluate the output got from each of the systems. The fluency check is done by *n*-gram analysis using the reference translations.

### 7.1. BLEU

We have used BLEU (bilingual evaluation understudy) to calculate the score of system output. BLEU is an IBM developed metric, which uses modified n-gram precision to compare the candidate translation against reference translations [16].

**Figure 3:**
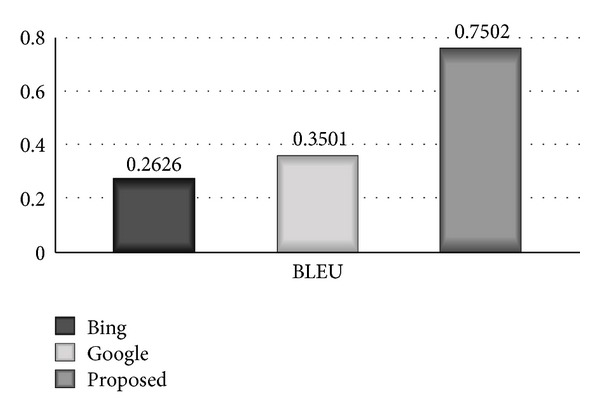
Comparative bar diagram between proposed system, Google, and Bing based on BLEU scale.

Comparative bar diagram between proposed system, Google, and Bing based on BLEU scale is shown in Figure 3. The bar diagram clearly shows that the proposed system has remarkably high accuracy of 0.7502 on BLEU scale, Bing has shown accuracy of 0.2626, and Google has shown 0.3501 accuracy on BLEU scale.

Calculate the *n*-gram resemblance by comparing the sentences. Then add the clipped *n*-gram counts for all the candidate sentences and divide by the number of candidate *n*-grams in the test sentence to calculate the precision score, *p*_*n*_, for the whole test sentence

(3) $$\begin{array}{c} p_{n}=\frac{\sum_{C\in \{\text{Candidates}\}}\sum_{n\text{-gram}\in C}\text{Coun}{\text{t}}_{\text{clip}}\left(n\text{-gram}\right)}{\sum_{C^{\prime }\in \{\text{Candidates}\}}\sum_{n\text{-}{\text{gram}}^{\prime }\in C^{\prime }}\text{Coun}{\text{t}}_{\text{clip}}\left(n\text{-}{\text{gram}}^{\prime }\right)}, \end{array}$$

where Count_clip_ = min (Count; Max Ref Count). In other words, one truncates each word's count.

Here *c* denotes length of the candidate translation and *r* denotes reference sentence length. Then calculate brevity penalty BP:

(4) $$\begin{array}{c} \text{BP}=\{\begin{array}{cc} 1 & \text{if}\;c>r\\ e^{\left(1-r/c\right)} & \text{if}\;c\leq r. \end{array} \end{array}$$

Then,

(5) $$\begin{array}{c} \text{BLEU}=\text{BP}\cdot \exp\left(\sum_{n=1}^{N}w_{n}\log\;p_{n}\right). \end{array}$$

### 7.2. NIST

**Figure 4:**
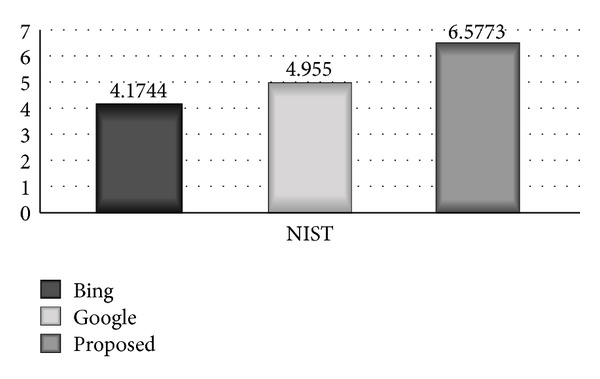
Comparative bar diagram between proposed system, Google, and Bing based on NIST scale.

Proposed by NIST (national institute of standard and technology), it reduces the effect of longer *N*-grams by using arithmetic mean over *N*-grams counts instead of geometric mean of cooccurrences over *N* [17].  Figure 4 shows the comparative bar diagram between proposed system, Google, and Bing based on NIST scale. The bar diagram clearly shows that the proposed system has remarkably high accuracy of 6.5773 on NIST scale, Bing has shown accuracy of 4.1744, and Google has shown 4.955 accuracy on NIST scale

(6) NISTscore=BPNIST∗PRECISIONNIST,PRECISIONNIST  =∑n=1N{∑all_w1⋯wn_that_co-occurInfo(w1⋯wn)∑all_w1⋯wn_in_hypo(1)},

where

(7) $$\begin{array}{c} \text{Info}\left(w_{1}\cdots w_{n}\right)=-\log_{2}\left(\frac{\text{Count}\left(w_{1}\cdots w_{n}\right)}{\text{Count}\left(w_{1}\cdots w_{n-1}\right)}\right), \end{array}$$

where Info weights more the words that are difficult to predict and count is computed over the full set of references; theoretically the precision range is having no limit

(8) $$\begin{array}{c} \text{B}{\text{P}}_{\text{NIST}}=\exp\left\{\beta *\log^{2}\left[mn\left(\frac{\text{LenHypo}}{\text{LenRef}},1\right)\right]\right\}, \end{array}$$

where LenHypo is total length of hypothesis and LenRef is average length of all references which does not depend on hypothesis.

### 7.3. ROUGE-L

**Figure 5:**
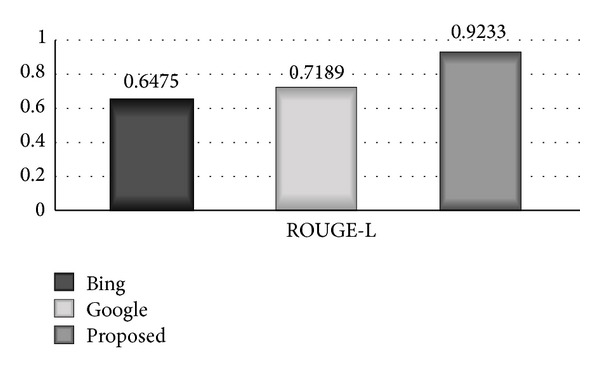
Comparative bar diagram among proposed system, Google, and Bing based on ROUGE-L scale.

ROUGE-L (recall-oriented understudy for gisting evaluation-longest common subsequence) calculates the sentence-to-sentence resemblance using the longest common substring among the candidate translation and reference translations. The longest common substring represents the similarity among two translations. *F*_lcs_ calculates the resemblance between two translations *X* of length *m* and *Y* of length *n*; *X* denotes reference translation and *Y* denotes candidate translation [18]. Comparative bar diagram between proposed system, Google, and Bing based on ROUGE-L scale is shown in Figure 5. The bar diagram clearly shows that the proposed system has remarkably high accuracy of 0.9233 on ROUGE-L scale, Bing has shown accuracy of 0.6475, and Google has shown 0.7189 accuracy on ROUGE-L scale

(9) $$\begin{array}{c} R_{\text{lcs}}=\frac{\text{LCS}\left(X,Y\right)}{m},\\ P_{\text{lcs}}=\frac{\text{LCS}\left(X,Y\right)}{n},\\ F_{\text{lcs}}=\frac{\left(1+\beta^{2}\right)R_{\text{lcs}}P_{\text{lcs}}}{R_{\text{lcs}}+\beta^{2}P_{\text{lcs}}}, \end{array}$$

where *P*_lcs_  is precision and  *R*_lcs_ is recall and LCS(*X*, *Y*) denotes the longest common substring of *X* and *Y*, and *β* = *P*_lcs_/*R*_lcs_ when ∂*F*_lcs_/∂*R*_lcs_ = ∂*F*_lcs_/∂*P*_lcs_

(10) $$\begin{array}{c} \text{Rouge-L}=\text{Harmonic Mean}\left(P_{\text{lcs}},R_{\text{lcs}}\right)=\frac{\left(2*P_{\text{lcs}}*R_{\text{lcs}}\right)}{\left(P_{\text{lcs}}+R_{\text{lcs}}\right)}. \end{array}$$

### 7.4. METEOR

**Figure 6:**
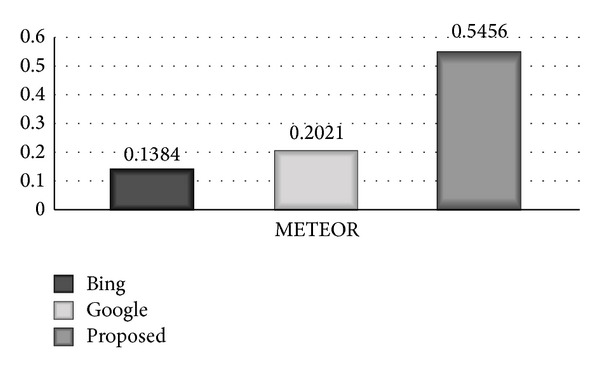
Comparative bar diagram among proposed system, Google, and Bing based on METEOR scale.

METEOR (metric for evaluation of translation with explicit ordering) is developed at Carnegie Mellon University.  Figure 6 shows comparative bar diagram between proposed system, Google, and Bing based on METEOR scale. The bar diagram clearly shows that the proposed system has remarkably high accuracy of 0.5456 on METEOR scale, Bing has shown accuracy of 0.1384, and Google has shown 0.2021 accuracy on METEOR scale.

The METEOR weighted harmonic mean of unigram precision (*P* = *m*/*w*_*t*_) and unigram recall (*R* = *m*/*w*_*r*_) used.

Here *m* denotes unigram matches, *w*_*t*_ denotes unigrams in candidate translation, and *w*_*r*_ is the reference translation. *F*_mean_ is calculated by combining the recall and precision via a harmonic mean that places equal weight on precision and recall as (*F*_mean_ = 2*PR*/(*P* + *R*)).

This measure is for congruity with respect to single word but for considering longer *n*-gram matches; a penalty *p* is calculated for the alignment as (*p* = 0.5(*c*/*u*_*m*_)^3^).

Here *c* denotes the number of chunks and *u*_*m*_ denotes the number of unigrams that have been mapped [19].

Final METEOR-score (M-score) can be calculated as follows:

(11) $$\begin{array}{c} \text{Meteor-score}=F_{\text{mean}}\left(1-p\right). \end{array}$$

Experiments confirm that the accuracy was achieved for machine translation based on quantum neural network, which is better than other bilingual translation methods.

## 8. Conclusion

In this work we have presented the quantum neural network approach for the problem of machine translation. It has demonstrated the reasonable accuracy on various scores. It may be noted that BLEU score achieved 0.7502, NIST score achieved 6.5773, ROUGE-L score achieved 0.9233, and METEOR score achieved 0.5456 accuracy. The accuracy of the proposed system is significantly higher in comparison with Google Translation, Bing Translation, and other existing approaches for Hindi to English machine translation. Accuracy of this system has been improved significantly by incorporating techniques for handling the unknown words using QNN. It is also shown above that it requires less training time than the neural network based MT systems.

## Figures and Tables

**Algorithm 1:**
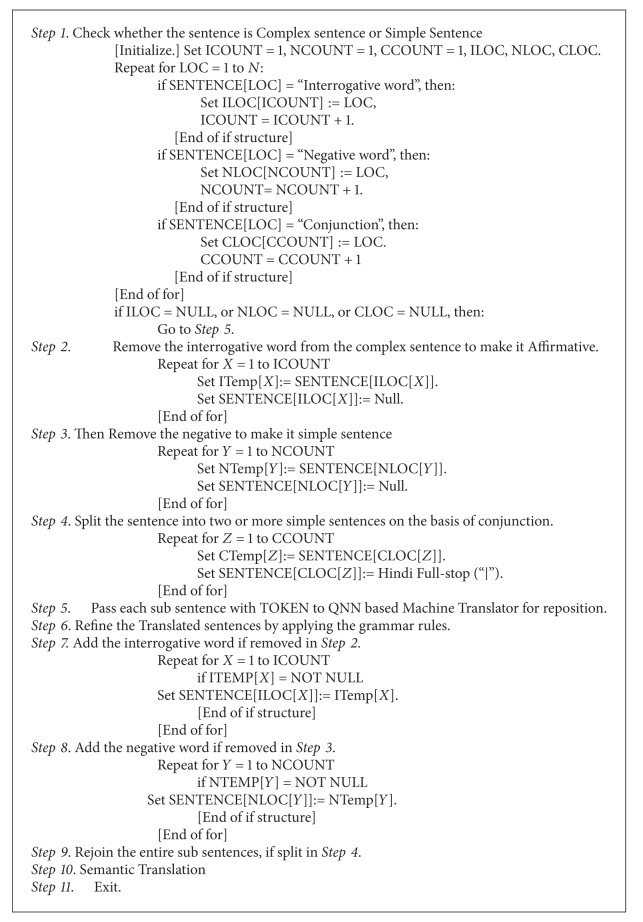


**Table 1:** Numeric codes for parts of speech.

Parts of speech (subclass)	Numeric code
Prenoun (PN)	.100
Noun-infinitive (Ni)	.101
Pronoun (PRO)	.102
Gerund (GER)	.103
Relative pronoun (RPRO)	.104
Postnoun (POSTN)	.105
Verb (V)	.110
Helping verb (HV)	.111
Adverb (ADV)	.112
Auxiliary verb (AUX)	.113
Interrogative (question word) (INT)	.120
Demonstrative words (DEM)	.121
Quantifier (QUAN)	.122
Article (A)	.123
Adjective (ADJ)	.130
Adjective-particle (ADJP)	.131
Number (N)	.132
Preposition (PRE)	.140
Postposition (POST)	.141
Punctuation (PUNC)	.150
Conjunction (CONJ)	.160
Interjection (INTER)	.170
Negative word (NE)	.180
Determiner (D)	.190
Idiom (I)	.200
Phrases (P)	.210
Unknown words (UW)	.220

**Table 2:** Comparison of performance measurement of MT system with QNN and traditional neural network.

S. No	Quantum interval (*θ*)	Epoch (iteration)	Training performance (MSE)	Validation performance (MSE)	Test performance (MSE)
1	*θ* = 0 is equivalent to ANN	20.04391	0.002027	0.002066	0.002075
2	0.25	15.36727	0.000185	0.000205	0.000203
3	0.5	15.47305	0.000248	0.000273	0.000273
4	0.75	15.28343	0.000231	0.000248	0.000252
**5**	1*	**15.48303**	**0.00025**	**0.00027**	**0.000272**
6	1.25	15.67066	0.000284	0.0003	0.0003
7	1.5	15.64271	0.000296	0.000318	0.000316
8	1.75	15.78643	0.000349	0.000374	0.000375
9	2	15.68663	0.000184	0.000204	0.000209
10	2.25	15.96607	0.000249	0.000273	0.000273
11	2.5	16.27345	0.000256	0.000281	0.000276
12	2.75	16.54092	0.00022	0.000242	0.000242
13	3	16.49301	0.000348	0.000361	0.00037
14	3.25	16.93214	0.000192	0.000214	0.000216
15	3.5	17.76248	0.000294	0.000315	0.000323
16	3.75	17.85429	0.000185	0.000202	0.000207
17	4	18.72056	0.000217	0.000237	0.00024

*For the value of *θ* = 1, the optimum trade-off of iteration and the minimum Error (MSE) achieved.
